# Effects of physical activity programs on sleep outcomes in older adults: a systematic review

**DOI:** 10.1186/s12966-020-0913-3

**Published:** 2020-02-05

**Authors:** J. Vanderlinden, F. Boen, J. G. Z. van Uffelen

**Affiliations:** 1 Department of Movement Sciences, KU Leuven, B-3000 Leuven, Belgium; 2 Department of Health Care, Odisee University College, Brussels, Belgium

**Keywords:** Sleep, Sleep quantity, Sleep quality, Physical activity, Exercise, Older adults, Aged, Elderly

## Abstract

**Background:**

One in two older adults report sleep problems, which not only cause fatigue, but also negatively affect general functioning, activities of daily living, and physical and mental health. Although it is known that physical activity is positively associated with sleep in older adults, the effects of physical activity programs on sleep in older adults has not been reviewed. The aim of this systematic review was to systematically review the effects of physical activity programs on sleep in generally healthy older adults aged 60+ years.

**Methods:**

Searches were performed in PubMed, Embase, Web of Science, SPORTDiscus, PEDro and CINAHL. The methodological quality of the included studies was rated using the ‘Quality Assessment Tool for Quantitative Studies’. Only studies of moderate and strong quality were included. This review was registered in PROSPERO (CRD42018094007).

**Results:**

Fourteen studies met the inclusion criteria (six randomised controlled trials and eight pretest-posttest studies). Of these studies, five were moderate and nine were strong quality studies. Mean age of study samples ranged from 64 to 76 years. Exercise programs included various activities aimed at improving mobility, endurance and strength. Intervention duration ranged from 2 weeks to 12 months. Eleven studies used subjective measures of sleep, two used objective measures and one used both. Sixteen different sleep outcomes were reported. All but one study, found at least one significant improvement on sleep outcomes. No significantly detrimental effects were reported. Effect sizes, calculated in ten studies, ranged from 0,34–1,55 and were substantial (≥0,8) in four studies.

**Conclusions:**

This systematic review suggests that exercise programs positively affect various aspects of sleep in generally healthy older adults. More specifically, moderate intensity exercise programs, with a frequency of three times per week and a duration of 12 weeks up to 6 months, showed the highest number of significant improvements in different sleep outcomes in older adults. Furthermore, programs that offered single exercise types, such as Baduanjin, Tai chi and the silver yoga program, or a combination of exercises showed the highest proportion of significant versus reported effects on sleep outcomes.

## Background

Ageing is associated with changes in sleep [1, 2]. When ageing, people spend more time in bed but less time asleep [3]. Sleep becomes less efficient and more disrupted. This goes along with decreases in slow wave sleep and increased early-morning awakenings [4–9].

The prevalence of sleep problems increases from the age of 65 years [10–12]. Approximately 50% of older adults suffer from difficulties in sleeping [13–15] of which up to 30% suffer from insomnia and 20% suffer from sleep apnea [11, 16]. Sleep problems in older adults can cause fatigue, daytime sleepiness and napping [17]. Sleep problems also affect general functioning, activities of daily living (ADL) and are associated with poorer quality of life, as well as cognitive and mental health issues [4, 18–23]. Furthermore, they are associated with an increase in functional impairments [24] and the development of cardiovascular disease [25, 26], metabolic syndrome, diabetes type 2 and obesity [27–30].

Treatment options for sleep problems include both pharmaceutical and non-pharmaceutical approaches [7]. Although pharmaceutical treatment options are often prescribed to older adults [31], they may cause side effects and are not always effective or safe in the long term [20, 32–35]. According to the American National Sleep Foundation (2019), non-pharmacological treatment options are the preferred first choice of treatment for sleep problems [36]. Pharmaceutical options should be prescribed after, or in combination with, a more durable non-pharmaceutical treatment. Non-pharmaceutical interventions can include cognitive behavioural therapy [16, 37–39], sleep hygiene advice, relaxation exercises [40–45] and physical activity [16, 46–50].

There is evidence for a beneficial effect of regular physical activity and exercise on sleep in adults in general [51], and in young [52, 53], middle aged [20, 54] and older adults specifically [20, 54–56]. Moreover, physical activity and exercise tend to be associated with decreased use of sleep medication [57–59].

Physical activity, taken regularly, may promote relaxation and energy expenditure in ways that is beneficial to initiating and maintaining sleep [11, 35, 60–62]. Therefore, using physical activity as a non-pharmaceutical treatment option for sleep problems could constitute an inexpensive, accessible and simple means of improving sleep in older adults [35].

Although various studies have examined the effects of physical activity on sleep in generally healthy older adults, the evidence has not yet been summarised. This constitutes a gap in the literature, given the age-related declines in physical activity and sleep, which are both important health indicators for successful ‘healthy ageing’ [63, 64]. Therefore, the aim of this paper is to systematically review current evidence on the effects of physical activity programs on sleep in generally healthy older adults.

## Methods

### Protocol

This review followed the preferred reporting items for systematic review protocols (PRISMA) [65]. The protocol was registered in PROSPERO in April 2018 (CRD42018094007; https://www.crd.york.ac.uk/PROSPERO/).

### Search strategy

Full bibliographic database searches were performed in June 2018 in six electronic databases (PubMed, Embase, Web of Science, SPORTDiscus, PEDro, CINAHL), using thesaurus and free terms for physical activity, exercise, sleep and ageing. The search strategy was developed in collaboration with an information specialist. The literature search was limited to studies that were published in English language and in peer-reviewed journals. There were no restrictions regarding publication date or country of publication of the articles. Full search strategies are available from the first author on request.

### Study eligibility criteria

Studies were eligible for inclusion when they examined the effects of physical activity programs or, more specifically, exercise programs, on sleep in older adults aged 60+ years. To be included in this review, studies had to meet the following criteria. (1) Population: generally healthy community-dwelling older adults or older adults in residential care aged 60+ years. In this review ‘generally healthy older adults’ refers to participants free from pre-existing major chronic diseases, such as cognitive or functional impairments, cardiovascular disorders, cancer, mental or psychiatric disorders, or sleep problems. Studies with study samples with an age range starting below 60 years, were only included if the minimum age was not lower than 50 years and the mean age was at least 60 years; (2) Intervention: interventions or programs which included physical activity or, more specifically, exercise. We excluded studies that reported acute effects of physical activity or exercise after just one session or associations between overall physical activity levels with sleep outcomes; (3) Outcome: objectively or subjectively measured sleep outcomes; (4) Design: we included intervention studies and (non-) randomised trials. Cross-sectional and qualitative studies, reviews, meta-analyses, and guidelines were excluded.

### Study selection

**Fig. 1:**
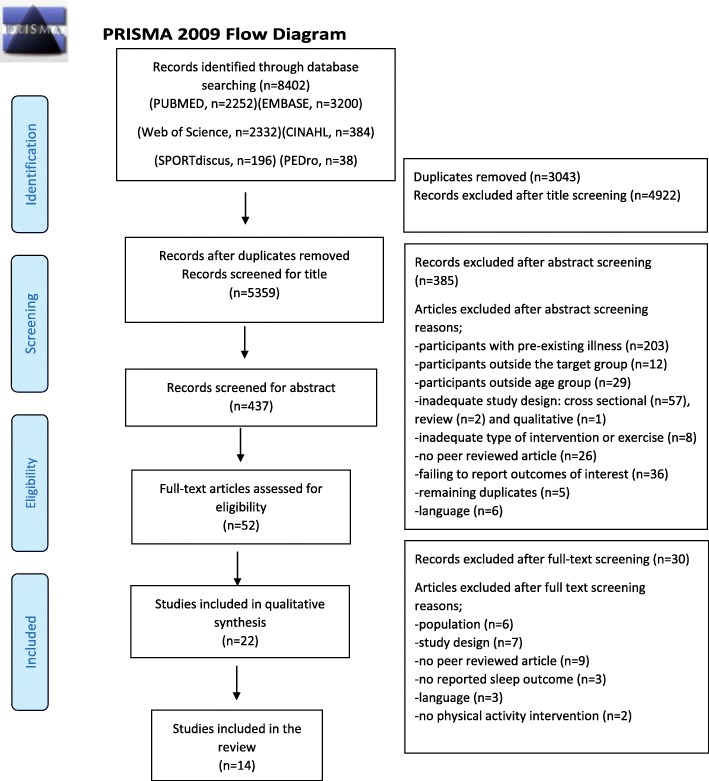
Search process and selection (PRISMA Flow diagram)

The search process and study selection were performed independently by two researchers. After loading all records of the different databases in Endnote (Version X8.1), duplicates were removed. Clearly irrelevant articles were excluded based on title screening. After abstract screening, full text of the remaining articles was retrieved and independently assessed for eligibility by two researchers. The references of articles identified through database searches were examined in order to identify any further potentially relevant studies. The search strategy is reported in a PRISMA flow chart (Fig. 1).

### Quality assessment

Study quality was assessed using the standardised tool “Effective Public Health Practice Project Quality Assessment Tool for Quantitative Studies”, recommended by the Cochrane Collaboration [66, 67]. This tool consists of eight items addressing selection bias, study design, confounders, blinding, data collection methods, withdrawals and dropouts, intervention integrity and analysis. Each item was rated as: ‘strong’, ‘moderate’ or ‘weak’. An item was considered ‘weak’ if the study failed to present a clear and transparent description [66, 67]. Studies without weak ratings on any of the eight items were labelled as ‘strong’. Studies with one weak rating were labelled as ‘moderate’ and two or more weak ratings resulted in an overall ‘weak’ study rating. Weak quality studies were excluded from further analysis in this review [66, 67].

The quality rating was independently performed by two researchers. Disagreements were resolved by discussion between the two raters. There was a strong inter-rater agreement (Cohens’ Kappa = 0.863, *p* < 0.001).

### Data synthesis

Details of physical activity programs and sleep outcomes were descriptively reported (Table 1). Most measures of sleep outcomes are combinations of sleep quality and quality, rather than measures of one single aspect of sleep. It was therefore not possible to separately report the effects on sleep quantity and quality. Effect sizes were calculated for all studies that reported relevant data (Table 2). Authors were contacted to provide further details if the data to calculate effect sizes were not included in the paper. A Cohen’s d > 0.80 was considered to indicate a substantial effect size [82].

**Table 1:** Sample and study characteristics

Article	Sample size	Age (range and mean)	Setting	Duration exercise program	Data collection	Intervention group	Control group	Sleep measurement
Stevenson and Topp (1990) [68]	*n* = 72 (intervention group: moderate intensity exercise *n* = 39, control group: low intensity exercise *n* = 33)	60-81y (mean age 63,9y)	community dwelling older adults	9 months	month 0 - month 4,5 - month 9	Supervised exercise session: stretching, cycling, slow walking and stretching, moderate intensity 60–70% HRR (3/week, 55–60 min)	low intensity exercise group as control (same exercise as intervention group, but lower intensity 30–40% HRR)	self-reported Likert type instrument developed by Topp (1986)
Naylor et al. (2000) [69]	*n* = 23 (intervention group *n* = 14, control group *n* = 9)	intervention group: 65-92y (mean age 75,2y); control group: 66-92y (mean age 71,2y)	homes or appartement complexes	14 days	day 0 - day 14	Light physical activity, stretching, cooling down stretching exercises (1x/day, 45 min)	usual daily activities	polysomnography
Frye et al. (2007) [70]	*n* = 74 (intervention group 1 Tai Chi n = 23, intervention group 2 low impact exercise *n* = 28 and control group *n* = 21)	52-82y (mean age 69,2y)	community dwelling older adults	12 weeks	week 0 - week 12	Tai Chi and low impact exercises (3x/week, 60 min)	usual daily activities	PSQI (only subscale sleep disturbances)
Chen and Tseng (2008) [71]	*n* = 14	60-86y (mean age 68,93y)	senior activity center	4 weeks	week 0 - week 4	Silver yoga exercise program (3x/week, 70 min)	no control group	PSQI
Chen et al. (2009) [72]	*n* = 128 (intervention group, *n* = 62, control group, *n* = 66)	60-75y (mean age 69,20y)	senior activity centres	6 months	month 0 - month 3 - month 6	Silver yoga exercise program (3x/week, 70 min)	waitlist control group, usual daily activities	PSQI
Chen et al. (2010) [73]	*n* = 55 (intervention *n* = 31 and control, n = 24)	(mean age 75,40y)	assisted living facility	6 months	month 0 - month 3 - month 6	Silver yoga exercise program (3x/week, 70 min)	usual daily activities	PSQI
Hosseini et al. (2011) [74]	*n* = 56 (intervention group *n* = 27 and control group *n* = 29)	60-83y (mean age 68,74y)	nursing home	12 weeks	week 0 - week 12	Tai Chi (3x/week,20–25 min)	usual daily activities	PSQI
Chen et al. (2012) [75]	*n* = 55 (intervention group *n* = 27, control group *n* = 28)	intervention group: 60-83y (mean age 71,75y)	rural community setting	12 weeks	week 0 - week 4 - week 8 - week 12	Baduanjin exercise program (3x/week, 30 min)	usual daily activities	PSQI
Nguyen and Kruse (2012) [76]	*n* = 96 (intervention group *n* = 48, control group *n* = 48)	60-79y (mean age 68,9y)	community dwelling older adults	6 months	month 0 - month 6 - follow up	Tai chi training (2x/week, 60 min)	usual daily activities	PSQI
Oudegeest-Sander et al. (2013) [77]	*n* = 21 (intervention group *n* = 11, young control group *n* = 10)	intervention group: (mean age 69y) young control group: (mean age 27y)	local community setting	12 months	month 0 - month 6 - month 12	Cycling exercise training, 70–85% HR (3/week, 45 min)	usual daily activities	accelerometers: Sesewear pro 3 armband SWA
Hariprasad et al. (2013) [78]	*n* = 87 (intervention group *n* = 44, control group *n* = 43)	intervention group: (mean age 75,74y) control group: (mean age 74,78y)	nursing home	6 months	month 0 - month 6	Yoga program (1x/dag, 60 min)	waitlist control	PSQI
Melancon et al. (2015) [79]	*n* = 13	57-70y (mean age 64y)	community dwelling older adults	16 weeks	week 0 - week 16	Aerobic exercise: progressively increased intensity brisk walking (3x/week, 45 min + 5–10 min warm-up and cool down)	waitlist control	(1) polysomnography and (2) PSQI
Chan and Chen (2016) [80]	*n* = 169 (intervention group, *n* = 84 and control group, *n* = 85)	65-87y (mean age 71,28y)	senior activity centres	6 months	month 0 - month 3 - month 6	Senior elastic band exercise program: warm-up, aerobic motion, static stretching (3x/week, 40 min)	usual daily activities	PSQI
Curi et al. (2018) [81]	*n* = 61 (intervention group, n = 31, control group, *n* = 30)	60-74y intervention group: (mean age 64,25y) control group: (mean age 63,75y)	senior activity center	16 weeks	week 0 - week 16	Pilates (1x/week, 60 min)	usual daily activities	PSQI

**Table 2:** Overview of results: subjective and objective sleep outcomes from different studies (studies are ordered from highest to lowest number of significant sleep outcomes)

	Chen et al. (2012) [75]	Chen et al. (2010) [73]	Chen et al. (2009) [72]	Frye, et al. (2007) [70]	Hosseini et al. (2011) [74]	Nguyen and Kruse (2012) [76]	Stevenson and Topp (1990) [68]	Chan and Chen (2016) [80]	Naylor et al. (2000) [69]	Curi et al. (2018) [81]	Melancon et al. (2015) [79]	Chen and Tseng (2008) [71]	Hariprasad et al. (2013) [78]	Oudegeest-Sander et al. (2013) [77]	#significant improved /#reported outcome
**Program characteristics**															
− frequency − intensity − time − duration − type	− 3*/week − moderate − 30 min − 12 weeks − Baduanjin	− 3*/week − moderate − 70 min − 6 months − silver yoga exercises	− 3*/week − moderate − 70 min − 6 months − silver yoga exercises	− 3*/week − low-to-moderate − 60 min − 12 weeks − tai chi, low impact exercises	− 3*/week − moderate − 20-25 min − 12 weeks − Tai chi	− 2*/week − moderate − 60 min − 6 months − Tai chi	− 3*/week − moderate − 55-60 min − 6 months − stretching, cycling, slow walking	− *3/week − low − 40 min − 6 months − elastic band exercises	−1*/day − low − 45 min − 2 weeks − stretching, cooling down	− 1*/week − moderate − 60 min − 16 weeks − Pilates	− 3*/week − moderate − 45-60 min − 16 weeks − brisk walking	− 3*/week − moderate − 70 min − 4 weeks − silver yoga exercises	− 1*/day − moderate − 60 min − 6 months − yoga	− 3*/week −vigorous − 45 min − 12 months − cycling	–
**Measures of sleep, effect size and study quality**															
(s)ubjective or (o)bjective	s	s	s	s	s	s	s	s	o	s	s/o	s	s	o	–
effect size*	1.55	1.01	0.86	0.35 (group 1) – 0.52 (group 2)	0.44	1.52	n	0.34	n	0.44	0.48	n	0.55	n	
study quality	strong	strong	strong	strong	moderate	strong	strong	moderate	moderate	strong	moderate	moderate	strong	strong	
**Sleep outcomes**															
difficulties falling back to sleep							x*								1*/1 (100%)
NREM sleep											x*				1*/1 (100%)
subjective sleep quantity							x*								1*/1 (100%)
PSQI total score	x*	x*	x*		x*	x*		x*		x*	x	x	x*		8*/10 (80%)
sleep latency	x*		x*				x*	x*	x	x*	x	x	x	x	5*/10 (50%)
sleep disturbances	x*	x*		x*				x		x	x	x*	x		4*/8 (50%)
wake time after sleep onset									x		x*				1*/2 (50%)
slow wave sleep									x*		x				1*/2 (50%)
stage 2 sleep									x*		x				1*/2 (50%)
total sleep time									x*					x	1*/2 (50%)
daytime dysfunction	x*	x*	x*					x		x		x	x		3*/7 (43%)
sleep duration	x*	x*						x*		x	x	x	x		3*/7 (43%)
sleep medication	x*							x		x*		x	x		2*/5 (40%)
sleep efficiency	x*	x*						x*		x	x	x	x	x	3*/8 (38%)
sleep quality	x*		x*				x	x	x	x		x	x		2*/8 (25%)
awakenings														x	0*/1 (0%)
#significant improved /#reported outcome	8*/8 (100%)	5*/5 (100%)	4*/4 (100%)	1*/1 (100%)	1*/1 (100%)	1*/1 (100%)	3*/4 (75%)	4*/8 (50%)	3*/6 (50%)	3*/8 (38%)	2*/9 (22%)	1*/8 (13%)	1*/8 (13%)	0*/4 (0%)	

Effect size underlined: Cohen’s d effect size > 80 = substantial; n = data to calculate effect size not available;

*Effect sizes are based on the main reported outcome in the individual study; these are indicated in bold in the table;

Sleep outcomes: x = measured outcome; x* = significant improved measure; no negative outcomes were reported;

*NREM* Non-rapid eye movement sleep; *PSQI* Pittsburgh Sleep Quality Index

## Results

### Study selection and quality rating

A total of 8402 potential studies were identified through electronic database searches. After removing 3043 duplicates, 5359 articles remained of which 4922 articles were excluded after title screening. Most articles were excluded in this phase because population or study design did not match the eligibility criteria. From the remaining 437 articles, 385 articles were excluded after abstract screening. The most frequent reasons for exclusion in this phase were: (1) population did not meet the inclusion criteria (*n* = 244); (2) study design did not meet the inclusion criteria (*n* = 94); (3) no sleep outcome (*n* = 36); (4) manuscript not in English language (*n* = 6) or (5) remaining duplicates (*n* = 5). After reading the full text of the remaining 52 articles, an additional 30 studies were excluded with following reasons; (1) didn’t include population of interest (*n* = 6); (2) cross sectional studies (*n* = 7); (3) no peer reviewed article (*n* = 9); (4) no sleep outcome (*n* = 3); (5) not written in English (*n* = 3) and (6) no physical activity program (*n* = 2), yielding 22 studies that met the inclusion criteria. Eight studies were weak quality studies and were therefore excluded. In the end, 14 studies were included in this review. Of these studies, five were of ‘moderate’ and nine were of ‘strong’ quality [66, 67] (Fig. 1).

### Sample and study characteristics

Study designs included pretest-posttest studies (*n* = 8) and randomised controlled trials (RCT’s) (*n* = 6). Two studies were published in the year 2000 or before, three studies between the years 2001–2010 and nine studies between the years 2011 and 2018. Studies were done in Asia (*n* = 7), North America (*n* = 4), South America (*n* = 1), Europe (*n* = 1) and the Middle-East (n = 1). Sample sizes ranged from 13 to 169 participants. Mean age of study samples ranged from 64 years to 76 years. Three studies only included females and one study only included males (Table 1).

### Physical activity and exercise programs

All 14 studies reported the effects of exercise programs. Intervention duration of the programs ranged from 12 weeks up to 12 months in the six RCT’s [72, 75–78, 81] and from 2 weeks up to 9 months in the eight pretest-posttest studies [68–71, 73, 74, 79, 80]. Session duration ranged from 20 to 70 min and session frequency ranged from once per week up to once daily. The majority of the interventions were supervised by certified instructors (*n* = 10). Out of the 14 studies, one study did not have a control group. The control groups of the remaining studies varied from controls that maintained their usual activities (*n* = 9), were waitlist controls (*n* = 3) or followed a lower intensity exercise program (*n* = 1) (Table 1).

### Effects on sleep outcomes

Sixteen different sleep outcomes were reported. There were no significantly detrimental effects reported (Table 2). For three sleep outcomes (difficulties falling back to sleep, non-rapid eye movement sleep (NREM) and subjective sleep quantity), 100% of the studies that reported this outcome, found positive significant effects. The Pittsburgh Sleep Quality Index (PSQI) total score was the most frequently assessed outcome. A significant beneficial effect was reported in eight out of the ten studies (80%) reporting PSQI total score.

For six sleep outcomes (sleep latency, sleep disturbances, wake time after sleep onset, slow wave sleep, stage 2 sleep and total sleep time), 50% of the studies that reported this outcome, found positive significant effects. For the remaining outcomes (daytime dysfunction, sleep duration, use of sleep medication, sleep efficiency and sleep quality, less than 50% of the studies found positive significant effects. There were no significant effects reported for the outcome: awakenings. Effect sizes for the main sleep outcome as reported by the individual studies (indicated in bold in Table 2) could be calculated for ten studies. Effect sizes ranged from 0,34-1,55. In four out of these ten studies [72, 73, 75, 76], the effect size was considered to be substantial (Cohen’s d > 0.80) (Table 2).

### Effects on subjective sleep outcomes

Twelve out of the fourteen included studies used subjective measures, of which eleven [70–76, 78–81] used the PSQI. The PSQI is a frequently used validated 19-item questionnaire, consisting of seven subscales (sleep quality, sleep latency, sleep duration, habitual sleep efficiency, sleep disturbances, use of sleep medication, and daytime dysfunction) to assess quality of sleep over 1 month. The total score ranges from 0 to 21 points, with higher scores indicating worse sleep quality. A cut-off threshold of five points is indicative of a poor-quality sleeper [83–85]. Of the studies using PSQI, ten reported the overall PSQI score. Subscales of PSQI were reported by some of these studies, i.e., sleep quality (*n* = 6), sleep latency (*n* = 7), sleep duration (*n* = 7), habitual sleep efficiency (*n* = 7), sleep disturbances (*n* = 8), use of sleep medication (*n* = 5), and daytime dysfunction (*n* = 7).

One study used a Likert-type instrument that focused on difficulties falling back to sleep, subjective sleep quantity, sleep latency and sleep quality [68].

Eight studies, five out of six RCT’s [72, 75, 76, 78, 81] and three out of eight pretest-posttest studies [71, 73, 74], showed significant improvements in **PSQI overall total score**. Four out of these eight studies [72, 73, 75, 76] were not only statistically significant, but also substantial in terms of effect size (Cohen’s d > 0.80; effect sizes respectively d = 0.86, 1.55, 1.52 and 1.01).

There was a significant improvement in **sleep latency** in five out of ten studies (50%) that measured this component of sleep; three RCT’s [72, 75, 81] and two pretest-posttest studies [68, 80]. **Sleep disturbances** were measured in eight studies and significantly reduced in one RCT [72] and three pretest-posttest studies [70, 71, 73] (50%). **Daytime dysfunction**, reported in seven studies, significantly improved in two RCT’s [72, 75] and in one pretest-posttest study [73] (43%). **Sleep duration**, measured in seven studies, significantly improved in three out of seven studies (43%); one RCT [75] and two pretest-posttest studies [73, 80].

Two RCT’s out of five studies (40%) reported a significant reduction in **the use of sleep medication** [75, 81]. **Sleep efficiency**, was measured in eight studies and significantly improved in three studies (38%); one RCT [75] and two pretest-posttest studies [73, 80]. **Sleep quality** was measured in eight studies and was significantly improved in two RCT’s out of these eight studies (25%) [72, 75].

The only study that used the Likert type scale [68] also reported significantly less experienced **difficulties falling back to sleep** after being awake during the night and significantly improved **subjective sleep quantity**.

### Effects on objective sleep outcomes

Three of the fourteen included studies used objective measures of sleep, two pretest-posttest studies used polysomnography [69, 79] and one RCT used accelerometry (Sensewear®) [77]. Polysomnography measures the different sleep stages [86–88]. Accelerometry measures sleep latency, total sleep time, sleep efficiency and the number of awakenings during sleep [89].

**NREM sleep** was only reported by one polysomnographic study and was significantly improved (100%) [79]. **Slow wave sleep and stage 2 sleep** were significantly improved in one of the two polysomnographic studies (50%) that measured these outcomes [69]. **Wake time after sleep onset**, reported by both polysomnographic studies, was significantly improved in only one study (50%) [79].

**Total sleep time** was reported in two studies; one RCT [77] and one pretest-posttest study [69], but was only found to be significantly improved in one study (50%) [69].

The only study that used accelerometry [77], showed no significant effects on the reported outcomes **sleep latency, total sleep time**, **sleep efficiency** and **awakenings** during the night. The outcome; awakenings was only reported by this study [77].

## Discussion

The aim of this study was to systematically review the literature on the effects of physical activity programs on sleep in generally healthy older adults aged 60+ years. Fourteen studies, six RCT’s and eight pretest-posttest studies, were included in this systematic review. These fourteen studies, nine strong quality and five moderate quality studies, described the effects of exercise programs. Sixteen different sleep outcomes were reported. For four of these outcomes there were significant beneficial effects in more than 50% of the studies that measured each of these outcomes. Effect sizes could be calculated in ten out of fourteen studies and were considered to be substantial in four studies.

A meta-analysis of Kelley and Kelley (2017) demonstrated the effects of exercise on sleep outcomes in adults of 18 up to 72 years. They concluded that exercise programs resulted in significant improvements in overall sleep quality, sleep quality and sleep latency, but not in sleep duration, efficiency, disturbance or daytime functioning [90]. The findings of the present systematic review in older adults seem to be in contrast to what is stated by Kelley and Kelley (2017), as at least one third of the studies that measured sleep duration, sleep efficiency and daytime functioning, found statistically significant improvements.

Looking at clinical study populations, previous studies indicated that exercise improved sleep quality, duration, efficiency and total sleep time [20, 91, 92]. In the present review, more than half of the studies which measured sleep duration and at least 50% of the studies that measured sleep efficiency, also found positive significant effects on these outcomes.

Two systematic reviews examined the effects of exercise on sleep in adults aged over 40 years with sleep problems [20, 92]. These reviews showed that exercise did not affect sleep duration, efficiency, disturbance and daytime functioning. However, the PSQI overall score increased, sleep latency improved and the use of sleep medication decreased [20]. The results of the first review are partly in line with the positive findings of the current review among older adults without sleep problems; more than half of the studies measuring PSQI overall score and half of the studies measuring sleep latency reported positive significant effects. In 40% of the studies, the use of sleep medication was significantly decreased. In contrast to these reviews, we also found positive significant effects on sleep disturbances in half of the studies, measuring this outcome. In at least one third of the studies, sleep duration, daytime dysfunction and sleep efficiency was also significantly improved. Discrepancies between the current review and reviews in adults with chronic diseases or sleep problems may be attributable to the fact that the latter populations experience lower sleep quality than generally healthy older adults, resulting in a lower overall PSQI score [16, 93]. Therefore, in order to achieve beneficial effects on more aspects of sleep, the underlying co-morbidities in people with chronic conditions or sleep problems should be addressed simultaneously [93].

There was a large variety between the included studies in this review in terms of design, quality and measurement methods. We will discuss this heterogeneity and link it to the differences in the results.

Firstly, from the literature it is known that studies with a stronger **study design** are more likely to report robust results [94]. However, despite the fact that the six RCT’s in this review had a methodologically stronger design than the eight pretest-posttest studies, the studies were not notably different in terms of the reported sleep outcomes or significant effects.

Secondly, **study quality** could also be associated with different study results [95]. In this review, the proportion between significant versus reported effects was notably higher in the nine strong quality studies compared to the five moderate quality studies. As six out of nine strong quality studies [68, 70, 72, 73, 75, 76] and only one out of five moderate quality studies [74] reported a proportion of ≥75% of significant versus reported outcomes. Additionally, four out of nine studies with strong quality [72, 73, 75, 76] reported substantial effect sizes, while none of the studies with moderate quality rating reported an effect size that exceeded a Cohen’s d of 0.80. Thus, methodologically stronger studies were more likely to report more significant effects and larger effect sizes.

Thirdly, **objective and subjective sleep measures** may represent different outcomes in sleep quantity and quality [84, 86–89, 96, 97] that are equally important in understanding sleep [87]. Objective measures focus specifically on sleep stage measuring, whereas subjective measures focus more on the perceived and experienced sleep outcomes [86–89]. In a study that examined the relationship between objective and subjective measures of sleep in healthy older adults, there were few and only modest associations [98]. This is in line with the findings from the only study in our review that combined objective and subjective sleep measures [79]. This study found significant improvements for two objectively measured sleep outcomes (NREM and wake time after sleep onset), but not for any subjective outcome [79]. One reason for this could be that gradual age-related changes in sleep may cause older adults to adapt their perception of sleep quality to the actual changes in sleep, while not recognising their disrupted sleep [83].

The studies in our review used objective and subjective sleep measurements. However, it is complicated to identify sleep outcomes as being purely sleep quantity or quality [83, 97, 99]. PSQI, the most frequently used measure of sleep in this review, is a subjective assessment developed to assess ‘sleep quality’ [99]. However, some PSQI subscales could also refer to sleep quantity [84, 87, 97]. For example, ‘sleep latency’ is a PSQI subscale composed of a numerical score (time) and a categorical score that indicates the subjective possibility to fall asleep within 30 min. The former score could be seen as sleep quantity (time indication), the latter score could be seen as sleep quality (subjective possibility to fall asleep). One subscale of PSQI can therefore have an ambiguous meaning. Therefore, it turned out to be very complicated and artificial to separate outcomes purely into sleep quantity or quality.

In what follows, we will examine the individual characteristics of the exercise programs.

### Frequency of exercise

Two former studies of O’Connor and Youngstedt (1995) and Dzierzewski et al. (2014) stated that ‘regular’ physical activity may be useful in improving sleep quality and reducing daytime sleepiness [62, 100]. In terms of frequency of exercise, several studies emphasize the importance of ‘regular’ exercise, however ‘regular’ is not further defined.

The majority of the programs in our review (i.e., ten out of fourteen studies), offered exercises at the frequency of three times per week. In six out of these ten studies [68, 70, 72–75] the proportion of significant versus reported sleep outcomes was more than 50% in terms of the following sleep outcomes; PSQI overall score, subjective sleep quantity and difficulties falling back to sleep.

Based on these findings, exercise programs with a frequency of three times per week, reported a higher proportion of significant beneficial sleep outcomes compared to exercise programs with the highest frequency (i.e. once daily) and the lowest frequency (i.e. once weekly).

### Intensity of exercise

Tse et al. (2015) showed that low intensity exercises in older adults might be most preferable because of better compliance, lower risk of injuries, and long-term sustainability [101]. Based on the findings in this review, six out of ten exercise programs with moderate intensity exercises [68, 72–76] reported a proportion of more than 50 % of significant versus reported sleep outcomes such as PSQI overall score, subjective sleep quantity and difficulties falling back to sleep, compared to the vigorous exercise program. Although only one study in our review examined the effects of a vigorous intensity exercise program, it reported no significant beneficial effects [77]. These findings indicate that moderate intensity exercise is most beneficial in terms of sleep outcomes. However, low and combined low-to-moderate exercise intensity is preferable above vigorous intensity exercise when it comes to improving sleep outcomes. This result is consistent with earlier findings that low intensity [102] and moderate intensity exercises [103] have positive and significant effects on sleep quality and its components in older adults [91].

### Time and duration of exercise

Based on the findings in this review, seven studies with a program duration of 12 weeks up to 6 months [68, 70, 72–76], irrespective of their session duration, reported the highest proportion (> 50%) of significant versus reported sleep outcomes. The three studies with a program duration of 12 weeks [70, 74, 75] showed a higher number of significant outcomes on PSQI overall score. Additionally, four out of six studies with a program duration of six months [68, 72, 73, 76] did not only show a higher number of significant outcomes on PSQI overall score, but also on difficulties falling back to sleep and subjective sleep quantity, compared to studies with a shorter [69, 71] or longer [77] program duration.

In addition to session and program duration, the timing of the sessions can also influence the effect of exercise on sleep. Morning exercise sessions can maximise and prolong deep sleep and may also help to reset the sleep wake cycle by raising body temperature slightly in contrast to exercise that is performed too close to bed time, as this can lead to difficulties falling asleep and interrupted sleep [104, 105]. Even exercise sessions of 10 min per day (such as walking, swimming or biking) can already improve sleep outcomes [106].

### Type of exercise

The American National Sleep Foundation suggests to implement daily aerobic exercise for at least 150 min per week in order to improve sleep and to battle insomnia [106, 107]. Additionally, strength training is suggested [108] in order to fall asleep faster and wake up less frequently throughout the night [109], and yoga exercises are primarily advised when stress is preventing people to fall asleep [110]. It seems that a combination of different types of exercise is most effective in improving different sleep outcomes simultaneously [109]. In our review, only three out of fourteen studies offered a combination of exercise types [68–70]. In two out of these three studies [68, 70] the reported proportion of significant versus reported sleep outcomes was more than 50 %. In the remaining study [69], the proportion of significant versus reported sleep outcomes was 50 %. These results are in line with the earlier finding that a combination of different types of exercise is more effective in improving different sleep outcomes [109].

Furthermore, single exercise types, such as Baduanjin and Tai chi reported a proportion of 100% in terms of significant versus reported sleep outcomes. The silver yoga program also reported a proportion of 100% significant versus reported sleep outcomes when performed during a period of 6 months. Single exercise types such as yoga, brisk walking, Pilates, cycling and resistance exercises reported a lower proportion of significant versus reported sleep outcomes.

### Social participation

The **social participation** of older adults could also bring about benefits for sleep. A former study showed that older adults with greater social participation slept better, experienced lower levels of wake time after sleep onset, fewer wake bouts and decreased sleep fragmentation [111]. The authors explain this benefit in the sense of belonging and social integration through shared time and engagement in joint exercise activities with members of the same group [111]. The included studies in the present review did not elaborate on social participation, nor did they control for it. However, it is worth considering these aspects when developing future physical activity or exercise programs for older adults, given the possible additional benefits for sleep outcomes [111, 112].

### Program setting

In our review, no study controlled for the setting of the program (e.g., indoor vs. outdoor) that might co-influence sleep outcomes. However, exercising outdoors during daytime hours significantly increases exposure to and absorption of natural sunlight (daily bright light) and therefore improves sleep outcomes [35, 105, 106]. Bright light is considered the strongest stimulus for the circadian rhythm [14] and it helps to reset the sleep wake cycle [105].

### Strengths and limitations of the study

A first strength is that an extensive literature search was performed in six prominent research databases, providing a broad range of literature from different research domains. A second strength is that only moderate to strong quality studies were included in this review and that the majority (nine out of fourteen studies) were strong quality studies.

A first limitation is that no meta-analysis could be performed because of high heterogeneity between studies. More specifically, although the majority of the studies used the PSQI, it was not possible to perform a meta-analysis on this measure as studies reported on different PSQI subscales. A second limitation is that we were only able to identify published papers in the databases [113]. We therefore cannot exclude the possibility of publication bias.

### Generalisability

The conclusions from this review apply to generally healthy older adults, rather than to older adults with specific chronic conditions or sleep problems. In terms of the terminology ‘older people’, there is no strict consensus when it comes to determine ‘older age’. Based on United Nations and World Health Organisation, both 60 and 65 years are considered to be age thresholds to refer to ‘older adults’ [64, 114, 115]. In this review, we used the mean age of 60 years as suggested by United Nations [114] and in previous work [116]. Male older adults and elders aged 75 and over were underrepresented. Thus, the conclusions of this review are most applicable to generally healthy older women up to age 75 years.

Although we set out to examine the effects of physical activity and exercise programs on sleep, we did not identify any intervention studies examining the effects of physical activity programs. Thus, the conclusions of this review apply specifically to exercise programs aimed at improving components of fitness, rather than more general physical activity programs aimed at increasing physical activity levels [50].

### Implications/future research

Firstly, the population of older adults above 75 years is growing rapidly, is less physically active and has a higher prevalence of sleep problems [2]. Also, the majority of the participants of the included studies were female participants. Therefore, upcoming studies should make a serious effort to include more male older adults above 75 years.

Secondly, the quality appraisal in this review revealed that confounders, blinding methods, withdrawals and dropout rates in the articles were mostly lacking or poorly described. As these are important factors related to internal and external validity, future studies should definitely describe these factors more transparently. Studies with solid and transparent descriptions of their methodology and protocol [117, 118] could facilitate the possibility to conduct meta-analyses. In order to allow more comparability between exercise programs, future protocols should also emphasize the frequency, the intensity, the time and the type of exercise. Furthermore, effects sizes and information about the setting, the organisation, the adherence and the presence of an exercise instructor should be described. Future studies are encouraged to invest in more robust and longitudinal study designs with follow-up as well as high quality qualitative studies [119, 120] to examine effects of exercise immediately after the program and over time [118, 121, 122].

Thirdly, subjective sleep outcomes are preferably measured by the use of validated questionnaires and objective sleep outcomes are preferably measured by polysomnography or accelerometry [86–89]. Objective and subjective assessments of sleep may relate to different outcomes. In order to provide a better understanding of effects of physical activity and exercise on all aspects of sleep and to help address sleep problems more effectively, future studies should incorporate both objective and subjective measures [87, 96].

## Conclusion

This systematic review included fourteen studies that examined the effects of exercise programs on sleep outcomes in generally healthy older adults. The findings provide directions for characteristics of exercise programs to optimally affect sleep in generally healthy older adults based on current knowledge. Based on the results of this review, moderate intensity exercise programs, with a frequency of three times per week and a program duration of 12 weeks up to 6 months, reported the highest number of significant effects on sleep outcomes in older adults. Additionally, interventions that offered single exercise types, such as Baduanjin, Tai chi and the silver yoga program, or a combination of exercises showed the highest proportion in terms of significant versus reported effects on sleep outcomes.

## Data Availability

The datasets used and/or analysed during the current study are available from the corresponding author on reasonable request.

## Abbreviations

ADL: Activities of daily living
EPHPP: Effective Public Health Practice Project Quality Assessment tool for quantitative studies
NREM: Non-rapid eye movement sleep
PA: Physical activity
PSQI: Pittsburgh Sleep Quality Index
RCT: Randomised controlled trial
